# Gaussian barebone mechanism and wormhole strategy enhanced moth flame optimization for global optimization and medical diagnostics

**DOI:** 10.1371/journal.pone.0317224

**Published:** 2025-01-16

**Authors:** Jingjing Ma, Zhifang Zhao, Lin Zhang

**Affiliations:** 1 Department of Medicine, Division of Gastroenterology, Third Ward, The First Medical Centre of Chinese PLA General Hospital, Haidian District, Beijing, China; 2 Department of Respiratory and Critical Care Medicine, The First Medical Centre of Chinese PLA General Hospital, Haidian District, Beijing, China; Torrens University Australia, AUSTRALIA

## Abstract

Moth Flame Optimization (MFO) is a swarm intelligence algorithm inspired by the nocturnal flight mode of moths, and it has been widely used in various fields due to its simple structure and high optimization efficiency. Nonetheless, a notable limitation is its susceptibility to local optimality because of the absence of a well-balanced exploitation and exploration phase. Hence, this paper introduces a novel enhanced MFO algorithm (BWEMFO) designed to improve algorithmic performance. This improvement is achieved by incorporating a Gaussian barebone mechanism, a wormhole strategy, and an elimination strategy into the MFO. To assess the effectiveness of BWEMFO, a series of comparison experiments is conducted, comparing it against conventional metaheuristic algorithms, advanced metaheuristic algorithms, and various MFO variants. The experimental results reveal a significant enhancement in both the convergence speed and the capability to escape local optima with the implementation of BWEMFO. The scalability of the algorithm is confirmed through benchmark functions. Employing BWEMFO, we optimize the kernel parameters of the kernel-limit learning machine, thereby crafting the BWEMFO-KELM methodology for medical diagnosis and prediction. Subsequently, BWEMFO-KELM undergoes diagnostic and predictive experimentation on three distinct medical datasets: the breast cancer dataset, colorectal cancer datasets, and mammographic dataset. Through comparative analysis against five alternative machine learning methodologies across four evaluation metrics, our experimental findings evince the superior diagnostic accuracy and reliability of the proposed BWEMFO-KELM model.

## 1. Introduction

Optimization refers to finding a better solution when decision-makers address a problem. With the progress of science, technology, and the development of human society, an increasing number of optimization problems are encountered in reality. This necessitates optimization algorithms to evolve and adapt to the growing needs[1,2]. Today, two main types of optimization algorithms exist: meta-heuristic algorithms and deterministic algorithms [3]. Deterministic are common and classical, primarily encompassing mathematical planning methods such as dynamic programming, branch and bound, and the conjugate gradient method, among others. These mathematical planning approaches typically necessitate information on the derivatives of the optimization problem [4] and are computationally complex, exhibiting suboptimal performance when confronted with problems of large scale and extensive data. Meta-heuristic algorithms exhibit superior performance when tackling large-scale complex problems. Drawing inspiration from the fields of biology, chemistry, and physics, metaheuristics treat the optimization problem as a black box, focusing solely on optimizing the output without regard to its specific definition. Iteratively adjusting to the problem’s output, metaheuristics ultimately converge to the optimal solution [5]. Furthermore, the global optimization and stochastic optimization properties of metaheuristic algorithms enable them to avoid getting stuck in local optimality, making them widely applicable in solving real-world optimization problems today.

Meta-heuristic algorithms are generally classified as swarm intelligence optimization algorithms and evolutionary algorithms. evolutionary algorithms draw inspiration from biological genetics and natural selection. Evolutionary algorithms generally involve three steps: selection crossover and mutation [6]. Well-known evolutionary algorithms include the differential evolution algorithm (DE) [7] and genetic algorithm (GA) [4,8]. The swarm intelligence optimization algorithm mainly simulates the behavior of various groups of organisms in nature to find the solution, such as the predatory behavior, pathfinding behavior and aggregation behavior of animals. Some classical swarm intelligence optimization algorithm are the ant colony optimizer (ACO) [9], particle swarm optimizer (PSO) [10], gray wolf optimizer (GWO) [11] and moth flame optimizer (MFO)[12]. In recent years, some novel population algorithms have been proposed. The slime mold algorithm [13] simulates the foraging behavior of slime mold. The algorithm is divided into three stages, which simulate exploration, wrapping and digestion of slime bacteria, and the different stages of the algorithm are adjusted by positive feedback weights to balance exploitation and exploration. The hunger game search algorithm [14] constructs a mathematical model that simulates behaviors and activities driven by hunger, assigns weights based on the evaluation value of individuals within each population, and later searches for the global optimal solution based on the population’s constant proximity to food. The colony predation algorithm (CPA) [15] simulates four hunting scenarios of scattering, surrounding, contacting companions and finding the next prey during predation.

Many algorithms demonstrate excellent performance and practical value, but the question arises: why design a multitude of different algorithms? The answer lies in the No Free Lunch (NFL) theorem [16], which posits that a single algorithm cannot universally excel across all problems. This theorem serves as creative motivation for the continuous development of new algorithms, recognizing the inherent diversity and complexity of optimization challenges.

Moth flame optimization (MFO) is a swarm optimization algorithm, which is inspired by the moth’s nocturnal flight navigation method, with a novel idea and simple structure. MFO has important applications in some fields, such as Allam et al. [17] in the study of polycrystalline silicon cells, choose MFO as a parameter optimizer, compare the three proposed diode models respectively, the experimental results show that the model after MFO optimized parameters has the minimum RMSE and MBE. Li et al. [18] proposed a hybrid power forecasting model using MFO optimization parameters. Although MFO has some practical applications and outperforms some other optimization algorithms, due to the simple structure and principle of MFO, the algorithm is cannot obtain the best global optimal solution when performing the search. In recent years, many researchers have improved MFO, proposed more efficient MFO improvement algorithms, and used them to solve various problems. Wang et al. [19] proposed a chaotic MFO improvement algorithm, called chaotic moth-flame optimization (CMFO). Chaos is an unstable state, and CMFO uses the chaotic mechanism in the initialization and population update of MFO. CMFO is combined with KELM [20] in practical applications, and CMFO is used for both parameter optimizations of KELM and feature selection of high-dimensional medical data, including breast cancer data and Parkinson’s disease data. The CMFO-KELM model was tested against the original MFO and genetic and particle swarm algorithms, and the experiments showed that CMFO outperformed the other comparison models. Sapre et al. [21] proposed a new algorithm by combining Cauchy mutation, evolution boundary constraint handling and opposition based learning (OBL) with MFO in order to enhance the performance of MFO and improve the global search capability. The algorithm outperforms other competing algorithms on all 18 benchmark functions. Hassanien et al. [22] used rough sets theory to improve MFO to fit feature selection task (MFORSFS) and applied the improved algorithm to automatic detection of tomato pests and diseases. The feature selection effectiveness of the algorithm was evaluated by the classification accuracy of branch vector machines, and the comparison experiments showed that MFORSFS outperformed GA and PSO, and the results showed that the algorithm has good advantages in terms of classification accuracy, recall and F-Score, and does not increase the execution time and number of features. Next, we will focus on efforts to improve the MFO aspects in order to be inspired by the comparison.

Compared with other studies, this study has carried out sufficient experiments and demonstration. The proposed algorithm is compared with other improved MFO algorithms and other well-known algorithms on 30 datum function. At the same time, the algorithm balance, diversity and standard engineering tests are carried out. Finally, the algorithm is used to optimize the kernel parameters of a kernel-limiting learning machine, building blocks for machine learning models for medical diagnosis and prediction. In general, the main contributions are as follows:

■ This study introduces an enhanced Moth Flame Optimization (MFO) algorithm, denoted as BWEMFO, through the integration of the Gaussian barebone mechanism, wormhole strategy, and elimination strategy.
■ In this study, a BWEMFO-KELM model for medical diagnosis and prediction was constructed using the kernel parameters of the proposed BWEMFO optimized kernel limit learning machine.
■ The effectiveness of BWEMFO is showcased through rigorous performance evaluations on the IEEE CEC 2017 function set. Comparative analyses are conducted against conventional optimizers, advanced optimizers, and various MFO variants. The proposed BWEMFO-KELM performs diagnostic and predictive experiments on three medical datasets: breast cancer dataset, colorectal cancer datasets and mammographic dataset. After comparing with other five machine learning methods on four evaluation metrics, the experimental results show that the proposed BWEMFO-KELM model has higher diagnostic accuracy and reliability.

The paper is structured as follows: The introduction (Section 1) outlines the motivation, background, and specific contributions. Following that, Section 2 introduces the original MFO. In Section 3, the introduced mechanism is described, and the proposed BWEMFO is detailed. Section 4 presents all the results and analysis. Section 5 discusses the advantages of BWEMFO. Finally, Section 6 concludes the paper.

## 2. Overview of the original MFO

Moth Flame Optimization (MFO) is an optimization inspired by the behavior of moths during their nocturnal flights. The algorithm mimics the way moths maintain a constant angle with the moon to ensure a straight flight. In the presence of an artificial flame simulating the moon, moths exhibit a spiral flight pattern as they maintain a specific angle with the flame, gradually approaching it. MFO abstracts this motion model observed in moths and flames, employing the spiral motion as a mechanism for updating the population in the optimization process. In mathematical modeling, the moth population is represented by the symbol *M*, with the vector position of *M* denoting the variable to be solved. The variable *m* represents the subject of the population search, as expressed in Eq 1.

$$M=\left[\begin{array}{cccc} m_{1,1} & m_{1,2} & \cdots & m_{1,d}\\ m_{2,1} & \ddots & \ddots & m_{2,d}\\ \vdots & \ddots & \ddots & \vdots \\ m_{n,1} & m_{n,2} & \cdots & m_{n,d} \end{array}\right]$$ (1)

where *m* represents each moth, *n* represents the number of moths, and *d* represents the dimensionality of the problem. The fitness values for evaluating the merits of the moths are recorded in the *OM* and the *OM* matrix is shown in Eq 2.


$$OM={\left[{{OM}_{1},OM}_{2},\cdots {,OM}_{n}\right]}^{T}$$ (2)

Corresponding to the moth matrix is the flame matrix *F*.*F* is used to store the better result in the corresponding M matrix. the *F* matrix is shown in Eq 3.


$$F=\left[\begin{array}{cccc} f_{1,1} & f_{1,2} & \cdots & f_{1,d}\\ f_{2,1} & \ddots & \ddots & f_{2,d}\\ \vdots & \ddots & \ddots & \vdots \\ f_{n,1} & f_{n,2} & \cdots & f_{n,d} \end{array}\right]$$ (3)

The most central algorithm update formula is shown in Eq 4.


$$S(M_{i},F_{j})=|F_{j}-M_{i}|∙e^{bt}∙\cos\left(2\pi t\right)+F_{j}$$ (4)

**Fig 1:**
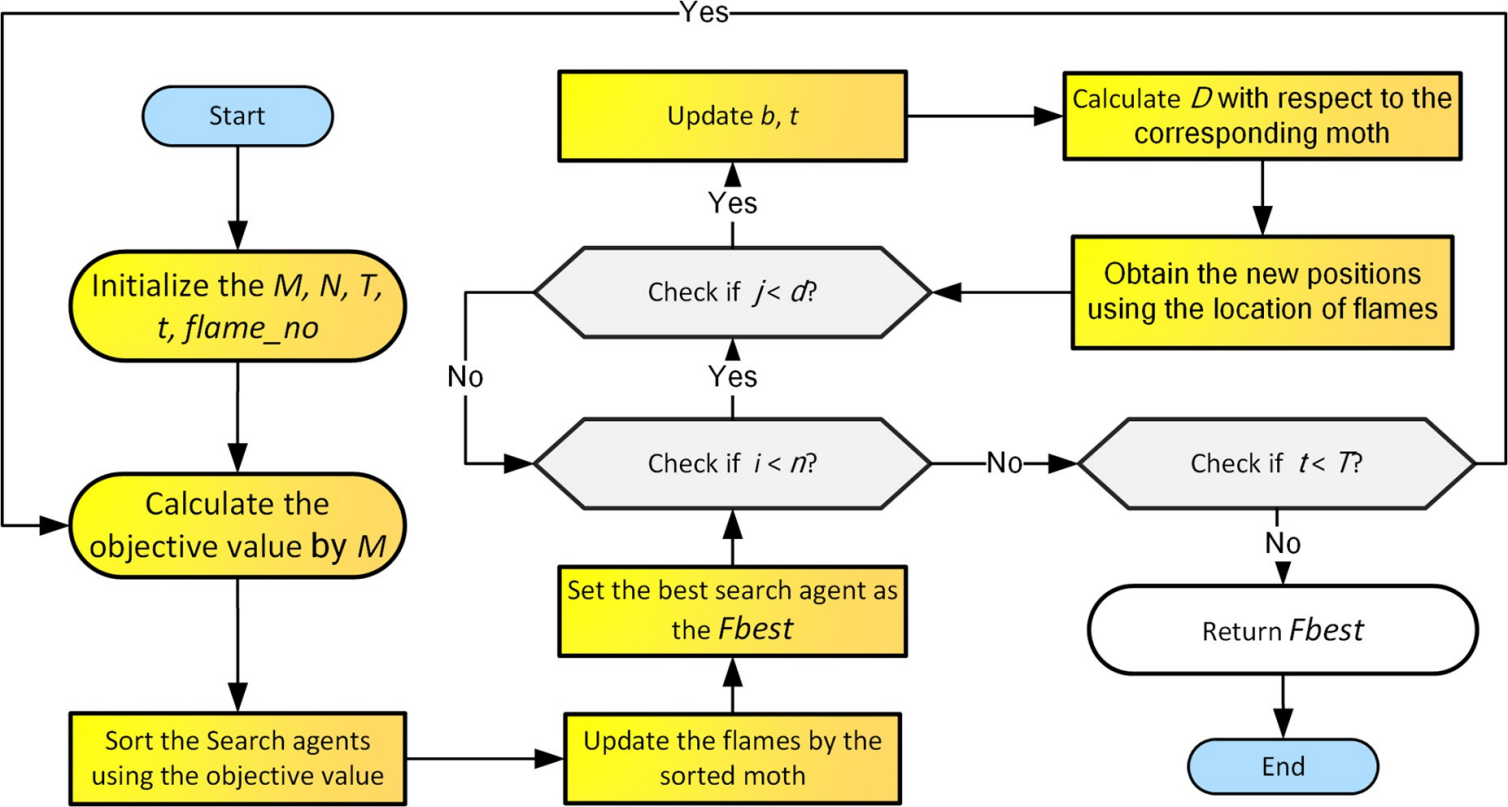
The flowchart of MFO.

Where *F*_*j*_ represents the *j*-flame, *M*_*i*_ represents the *i*-moth, |*F*_*j*_−*M*_*i*_| represents the distance between *F*_*j*_ and the corresponding *M*_*i*_.*b* is the logarithmic helix number. *t*∈[−1,1]. When t = 1, the moth flies in the direction away from the flame. When t = -1, the moth flies in the direction close to the flame. The spiral shape of the search shows that the population can search the solution around the flame more adequately in the search space. The flowchart for MFO is shown in Fig 1. In Fig 1, *M* represents the moth population. *N* represents the number of moth individuals within the population. *T* represents the maximum number of iterations. t represents the current number of iterations. *D* represents the distance between the moths and their corresponding flames, which are used to generate new individual moths. j represents the dimension currently being updated.

## 3. Proposed BWEMFO

Despite finding applications across various fields, MFO faces limitations in meeting the demands of current optimization problems due to the growing complexity of problems and the continual introduction of new algorithms. The simplicity of MFO’s population updating mechanism proves insufficient for the thorough exploration and exploitation of the search space. Consequently, this paper suggests the incorporation of three additional mechanisms into MFO to enhance its search capability.

### 3.1 Gaussian barebone mechanism (GB)

The Gaussian barebone (GB) mechanism was first proposed by Kennedy to construct the barebones PSO algorithm (BBPSO) [23]. BBPSO is an improved algorithm of PSO. Inspired by PSO, the algorithm ignores the velocity properties of particles and obtains the next iteration through gaussian distribution. BBPSO has obvious compact characteristics and nonparametric. In BBPSO, the particle position update formula is replaced with a formula containing gaussian distribution. As shown in Formula 5.

$$X_{i,j}^{t+1}=N(\frac{{pbest}_{i,j}^{t}+gbest}{2},|{pbest}_{i,j}^{t}-gbest|)$$ (5)

where $X_{i,j}^{t+1}$ represents the position of the particle, *i* represents the *i*-*th* particle, *j* represents the *j*-dimension of the *i*-*th* particle, *t*+1 represents the *t*+1 iteration process, ${pbest}_{i,j}^{t}$ represents the current optimal position of the particle, and *t* represents the *t*-iteration process. *gbest* represents the global optimal solution in the group search process, and *N* represents the Gaussian normal distribution.

The GB mechanism not only adopts the Gaussian distribution position mutation method in BBPSO, but also adds the branch strategy based on DE to balance the particle updating process [24]. The total updating formula is shown in Formula 6.

$$X_{i,j}^{t+1}=\left\{ \begin{array}{c} N(\frac{{pbest}_{i,j}^{t}+gbest}{2},\left|{pbest}_{i,j}^{t}-gbest\right|)\;rand<CR\\ {pbest}_{i,j}^{t}+k\times (X_{k1,j}^{t}-X_{k2,j}^{t})\;rand\geq CR \end{array}\right.$$ (6)

where *k* and *rand* are random numbers between 0 and 1, *CR* is used to control different update strategies, which is set to 0.9 in this study. *k*1 and *k*2 are random numbers in set 1-*N*.

### 3.2 The wormhole strategy (WS)

The wormhole strategy (WS) is derived from Multi-Verse Optimizer (MVO)[25], which is inspired by the concepts of white hole, black hole and wormhole in the universe. The three concepts are abstracted into mathematical models for the exploration, exploitation and local search of comfort. Inspired by MVO algorithm, WS is applied in MFO algorithm to jump out of local optimization. WS contains two main adaptive parameters WEP and TDR. WEP is mainly used to select the update method. TDR determines the weight of the current solution. WS also relies on the current optimal solution. Formulas 7–9 shows the update mechanism of WS in detail.

$$\vec{X_{i}}(t+1)=\left\{ \begin{array}{c} \{\begin{array}{c} \vec{X_{a}}+TDR\times ((UB-LB)\times r_{5}+LB),r_{4}<0.5\\ \vec{X_{a}}-TDR\times ((UB-LB)\times r_{5}+LB),r_{4}\geq 0.5 \end{array},WEP<r_{3}\\ \vec{X_{i-1}},\;WEP\geq r_{3} \end{array}\right.$$ (7)

$$WEP={WEP}_{min}\times FEs\times \left(\frac{{{WEP}_{max}-WEP}_{min}}{MaxFEs}\right)$$ (8)

$$TDR=1-\frac{{FEs}^{\frac{1}{p}}}{{MaxFEs}^{\frac{1}{p}}}$$ (9)

where *p* is set to 6 in the experiment to control the local search of the algorithm. *WEP*_*min*_ is set to 0.2 in the experiment, *WEP*_*max*_ is set to 1, and *WEP*_*min*_ and *WEP*_*max*_ are used to control the size of WEP value. *r*_3_,*r*_4_ and *r*_5_∈ (0,1], are random number.

### 3.3 The elimination strategy (ES)

The elimination strategy (ES) is mainly used to avoid local optimization [26]. Compared with other technologies, ES mainly acts on the last 5% of the elimination population. The main update formula of ES and the parameters used are shown in Formulas 10–12. Yang et al. [26] added ES and other mechanisms to the GWO and proposed a new BSWEGWO. BSWEGWO not only outperforms other comparison algorithms in benchmark function testing, but also shows better feature selection performance in public medical data sets.

$$\vec{X_{cencer}}=\frac{\vec{X_{a}}+\vec{X_{\beta }}}{2}$$ (10)

$$Dia=\sqrt{\left\|\vec{X_{a}}-\vec{X_{\beta }}\right\|}$$ (11)

$$\vec{X_{i}}=\left\{ \begin{array}{l} \vec{X_{center}}+rand\times Dia,k>0.45\\ \vec{X_{center}}\times k,\;k<0.45 \end{array}\right.$$ (12)

Where *k*∈(0,1), is a random number. $\vec{X_{a}}$ and $\vec{X_{\beta }}$ are candidate solutions of the same dimension of the first two individuals in the population.

### 3.4 The proposed BWEMFO

**Fig 2:**
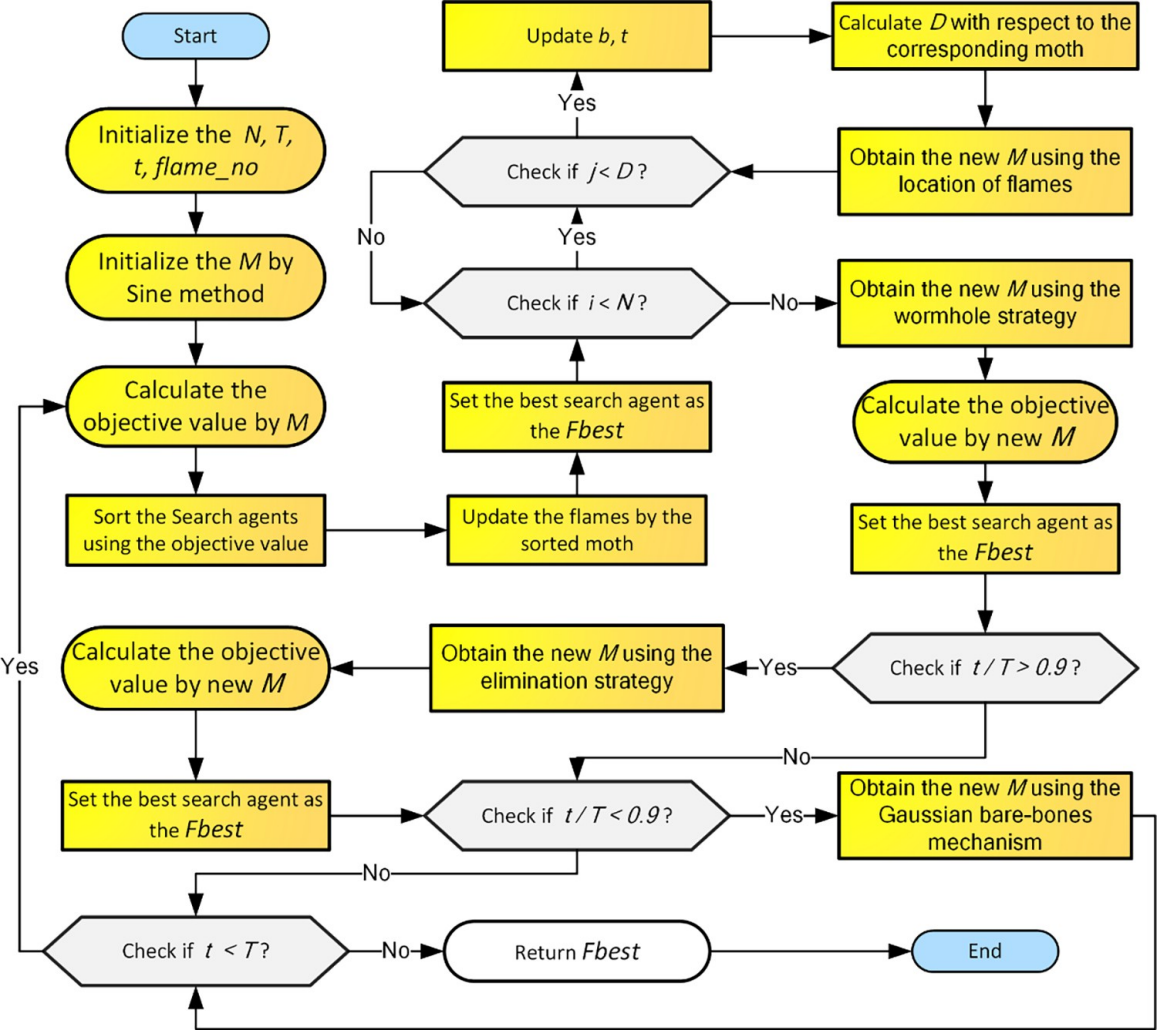
Flowchart of BWEMFO.

This section adds mechanisms mentioned above to the MFO. BWEMFO first initializes the moth population according to the set parameters. Starting the algorithm iteration, after the spiral update operator of the original MFO, the algorithm updates the population by wormhole strategy and elimination strategy, respectively, and updates the optimal solution. After that the population is updated again by Gaussian barebone mechanism for the next iteration. The flowchart of BWEMFO is shown in Fig 2. In Fig 2, *M* represents the moth population. *N* represents the number of moth individuals within the population. *T* represents the maximum number of iterations. t represents the current number of iterations. *D* represents the distance between the moths and their corresponding flames, which are used to generate new individual moths. In BWEMFO, we employed sine population initialization method during the population initialization phase. Sine initialization method is a method based on the sine function, designed to generate a more uniformly distributed population of initial solutions while enhancing the algorithm’s global search capability. By leveraging the periodic and nonlinear characteristics of the sine function, this method can effectively cover multiple regions of the search space, prevent solutions from being concentrated in specific areas, and improve the diversity of the population. The sine initialization method generates the initial positions of individuals using the sine function, as defined by the following equation:

$$X_{i,j}=X_{min,j}+(X_{max,j}-X_{min,j})\times \sin\left(r\times \pi \right),$$ (13)

where *r* is a random number within the range (0, 1), and sin(*r*×*π*) maps the random number distribution into a nonlinear distribution through the sine function.

Fig 2 shows the process of BWEMFO. The time complexity of BWEMFO is determined by the initialization, GB mechanisms, WS mechanisms, ES mechanisms and iterative updates of the MFO. The main influencing parameters are the iterations (*T*), the population size (*n*) and the dimension (*d*). Therefore, the time complexity of BWEMFO is *O*(BWEMFO) = *O*(initialization) + *O(*population update) + *O*(WS) + *O*(ES)+ *O*(GB) ≈ *O(n*×*d*) + *O*(*n*×*d*×*T*) + *O*(*n*) + *O*(*n*+*d*) + *O*(*n*×*d*) ≈ *O*(*T*×*d*×*n*).

## 4. Experimental results and analysis

The quality of BWEMFO’s performance will be strictly evaluated in several aspects. On the 30 functions of IEEE CEC 2017 [27], BWEMFO will be compared with traditional metaheuristics, variants of MFO, and improved metaheuristics. IEEE CEC 2017 test functions are often used to test the performance of comparative optimization algorithms. There are 30 functions, which are divided into single-peak, mixed, combined and multi-peak functions. The parameter settings of the algorithm in the experiment are shown in Table 1. In comparative studies of swarm intelligence optimization algorithms, population size is a crucial parameter. A population size of 30 is commonly adopted, and its selection can be justified from several perspectives. First, many classic studies recommend a default population size of 30. For instance, Kennedy and Eberhart, in their original work on Particle Swarm Optimization (PSO), suggested a population size between 20 and 50, with 30 being a frequently used value. Similarly, early experiments on other algorithms, such as Genetic Algorithm (GA), often employed 30 as a standard benchmark. Second, a smaller population size (e.g., 10–20) may lead to insufficient global search capability, increasing the likelihood of premature convergence to local optima. Moreover, limited diversity among individuals can hinder effective exploration of the search space.

**Table 1:** Parameters.

Population size	Dimension	Maximum evaluation
30	30	300000

### 4.1. Influence of the three mechanisms

The BWEMFO comprises a foundational MFO and three enhanced mechanisms, namely GB (Gaussian barebone), WS (Wormhole Strategy), and ES (Elimination Strategy). In this section, we aim to assess the impact of these three mechanisms on MFO and explore their individual contributions to the enhancement of MFO. Crucially, we will illustrate that the three mechanisms in BWEMFO synergistically reinforce each other, emphasizing that the absence of any one mechanism prevents achieving the optimal performance of BWEMFO.

Table 2 records the mechanisms for adding different improved MFO algorithms, namely BWEMFO, GBMFO, WSMFO, ESMFO, WSGBMFO, ESGBMFO, WSESMFO, and MFO. 1 in the table represents participating in the mechanism. 0 represents that no mechanism has been added. From Table 2, it can be seen that BWEMFO has added three mechanisms. GBMFO has added GB and ES. WSMFO has added WS and ES. ESMFO has added ES. WSGBMFO has added GB and WS. ESGBMFO has added ED and GB. WSESMFO has added WS and ES. MFO has not added any mechanism.

**Table 2:** Various MFOs from three mechanisms.

Algorithm	GB	WS	ES
BWEMFO	1	1	1
GBMFO	1	0	0
WSMFO	0	1	0
ESMFO	0	0	1
WSGBMFO	1	1	0
ESGBMFO	1	0	1
WSESMFO	0	1	1
MFO	0	0	0

Table 3 presents the experimental results of multi mechanism analysis for BWEMFO. ’Rank’ signifies the algorithm’s position, ’AVG’ represents its average ranking, and the symbols ’+/-/ = ’ denote whether the BWEMFO algorithm outperforms, underperforms, or equals other algorithms. This comparison is determined by the Wilcoxon rank sum test. When the p-value is less than 0.05, it can be considered that the results of BWEMFO are significantly better than other methods. The ranking is calculated through the Friedman test.

**Table 3:** Experimental results of multi mechanism analysis for BWEMFO.

Algorithm	Rank	+/ = /-	AVG
BWEMFO	1	~	1.4667	
GBMFO	6	28/0/2	5.9333	
WSMFO	2	27/3/0	2.4333	
ESMFO	5	30/0/0	5.9000	
WSGBMFO	4	29/1/0	3.8667	
ESGBMFO	7	30/0/0	6.2333	
WSESMFO	3	28/1/1	2.5667	
MFO	8	30/0/0	7.8333	

From Table 3, the ranking of the 8 variants from good to bad is BWEMFO> WSMFO> WSESMFO> WSGBMFO> ESMFO> GBMFO> ESGBMFO> MFOG. BWEMFO ranked first among all variants. All variants have better performance than the original MFO, indicating that all three improvement mechanisms have an enhancing effect on MFO. WSMFO ranks second, indicating that the WS mechanism has greatly improved MFO, but with the addition of ES and GB mechanisms, BWEMFO has achieved even greater improvement.

### 4.2. Scalability test

To assess the performance of BWEMFO across various dimensions, experiments were conducted on the IEEE CEC 2017 functions. The original MFO was also applied in dimensions of 50, 100, and the results are presented in Table 4. The findings indicate that the solution quality of MFO diminishes with increasing dimensionality. In contrast, BWEMFO consistently achieves superior and more stable results, showcasing its robust scalability. The proposed BWEMFO demonstrates the capability to handle high-dimensional problems stably and efficiently.

**Table 4:** Multidimensional test results of BWEMFO and MFO on IEEE CEC 2017 test functions.

F	Method	Dim = 50		Dim = 100	
		Avg	Std	Avg	Std
F1	BWEMFO	8.2753E+03	7.0944E+03	1.0337E+04	8.0945E+03
	MFO	1.1124E+10	8.0067E+09	9.8629E+09	6.7814E+09
F2	BWEMFO	1.7549E+04	3.7468E+04	2.6181E+02	1.0358E+02
	MFO	7.5744E+37	3.6493E+38	4.9823E+39	2.6531E+40
F3	BWEMFO	3.0043E+02	1.5734E-01	3.0003E+02	2.2605E-02
	MFO	1.0595E+05	8.4383E+04	1.0847E+05	7.8030E+04
F4	BWEMFO	4.8915E+02	6.6004E+00	4.9089E+02	9.2982E+00
	MFO	1.1225E+03	5.2729E+02	1.2966E+03	7.4276E+02
F5	BWEMFO	5.7767E+02	1.9640E+01	5.8253E+02	2.5918E+01
	MFO	7.0723E+02	4.5802E+01	7.0395E+02	5.0490E+01
F6	BWEMFO	6.0477E+02	3.2966E+00	6.0450E+02	3.7643E+00
	MFO	6.3730E+02	1.0058E+01	6.4008E+02	1.1230E+01
F7	BWEMFO	8.3927E+02	3.7160E+01	8.4843E+02	4.7558E+01
	MFO	1.1377E+03	1.8594E+02	1.1748E+03	1.7898E+02
F8	BWEMFO	8.9077E+02	2.7311E+01	8.8541E+02	2.5116E+01
	MFO	1.0178E+03	4.3218E+01	1.0079E+03	4.3189E+01
F9	BWEMFO	1.5132E+03	5.9235E+02	1.6346E+03	6.0120E+02
	MFO	6.9109E+03	1.5555E+03	7.0692E+03	1.6579E+03
F10	BWEMFO	4.0615E+03	6.8931E+02	3.9292E+03	6.5200E+02
	MFO	5.2328E+03	7.9528E+02	5.3884E+03	6.3574E+02
F11	BWEMFO	1.3234E+03	1.1053E+02	1.2769E+03	6.9511E+01
	MFO	4.0496E+03	3.9363E+03	4.5657E+03	3.9670E+03
F12	BWEMFO	2.0231E+06	2.2725E+06	1.4871E+06	1.7994E+06
	MFO	3.6651E+08	7.3712E+08	2.5586E+08	4.1951E+08
F13	BWEMFO	2.9565E+04	2.4603E+04	2.1866E+04	2.4505E+04
	MFO	3.2447E+08	1.0992E+09	4.8526E+07	2.4761E+08
F14	BWEMFO	1.4216E+04	6.0107E+03	7.9768E+03	4.3086E+03
	MFO	3.7407E+05	1.7399E+06	8.4840E+04	1.2309E+05
F15	BWEMFO	3.1398E+03	2.1856E+03	2.7602E+03	1.4491E+03
	MFO	4.2380E+04	3.3815E+04	5.2375E+04	5.6649E+04
F16	BWEMFO	2.2946E+03	3.1224E+02	2.2602E+03	2.9586E+02
	MFO	3.0644E+03	3.9235E+02	3.0985E+03	3.3298E+02
F17	BWEMFO	2.1081E+03	1.7375E+02	2.0712E+03	1.6191E+02
	MFO	2.5476E+03	2.5501E+02	2.4246E+03	2.8782E+02
F18	BWEMFO	2.1855E+05	1.3774E+05	2.1169E+05	1.8710E+05
	MFO	5.0282E+06	1.1993E+07	4.1269E+06	8.7514E+06
F19	BWEMFO	4.6762E+03	3.6687E+03	5.1788E+03	3.3005E+03
	MFO	2.2006E+07	4.9821E+07	1.1191E+07	3.7059E+07
F20	BWEMFO	2.3645E+03	2.1628E+02	2.2839E+03	2.1740E+02
	MFO	2.7264E+03	2.4560E+02	2.7424E+03	2.0682E+02
F21	BWEMFO	2.3691E+03	2.0625E+01	2.3806E+03	2.1708E+01
	MFO	2.5155E+03	4.5327E+01	2.5029E+03	4.3103E+01
F22	BWEMFO	2.3004E+03	1.0298E+00	2.3005E+03	1.0919E+00
	MFO	6.7320E+03	1.3434E+03	6.3348E+03	1.6372E+03
F23	BWEMFO	2.7364E+03	1.9155E+01	2.7364E+03	2.2115E+01
	MFO	2.8302E+03	4.3075E+01	2.8312E+03	3.1201E+01
F24	BWEMFO	2.8896E+03	1.9365E+01	2.8945E+03	2.3223E+01
	MFO	2.9899E+03	3.4766E+01	2.9908E+03	3.7779E+01
F25	BWEMFO	2.8925E+03	1.4136E+01	2.8897E+03	7.5555E+00
	MFO	3.2987E+03	4.4237E+02	3.2271E+03	3.2552E+02
F26	BWEMFO	4.3836E+03	6.4467E+02	4.4953E+03	6.0165E+02
	MFO	5.8871E+03	6.4013E+02	5.9024E+03	4.5514E+02
F27	BWEMFO	3.2181E+03	1.1886E+01	3.2182E+03	1.2502E+01
	MFO	3.2608E+03	3.5570E+01	3.2606E+03	3.7783E+01
F28	BWEMFO	3.2363E+03	3.3672E+01	3.2443E+03	3.6760E+01
	MFO	4.2998E+03	9.7295E+02	4.0483E+03	8.1914E+02
F29	BWEMFO	3.6333E+03	1.8424E+02	3.6276E+03	1.4199E+02
	MFO	4.0776E+03	2.6806E+02	4.2446E+03	2.1681E+02
F30	BWEMFO	1.4537E+04	6.4731E+03	1.1771E+04	4.8698E+03
	MFO	1.8453E+06	4.0635E+06	1.4433E+06	2.6440E+06
+/ = /-		30/0/0		30/0/0	

### 4.3. Comparison with other algorithms

The proposed BWEMFO was compared with eleven metaheuristic, which include CCWFO [28], QCSCA [29], WEMFO [30], CMFO [31], CCMWOA [32], SCADE [33], CGSCA[34], BA [35], MFO [12], SMA [13] and PSO [36]. The comparative experiments established uniform conditions for all algorithms, ensuring experimental fairness. In Table 5, we present the experimental outcomes of BWEMFO compared to a conventional algorithm across 30 functions.

**Table 5:** Comparative experimental ranking results of BWEMFO and other famous optimization methods on IEEE CEC 2017 test functions.

Algorithm	Rank	+/ = /-	AVG
BWEMFO	1	~	2.1311	
QCSCA	3	18/4/8	3.4333	
CCWFO	2	17/4/9	2.7667	
WEMFO	5	24/0/6	5.2300	
CMFO	8	29/0/1	7.5856	
CCMWOA	12	30/0/0	10.3944	
SCADE	11	30/0/0	10.2211	
CGSCA	10	30/0/0	9.3211	
BA	7	23/5/2	7.3922	
MFO	9	30/0/0	8.6044	
SMA	4	20/1/9	4.8666	
PSO	6	23/3/4	6.3900	

As depicted in Table 5, BWEMFO secured the top ranking among the 12 conventional algorithms across 30 test functions. The second ranked algorithm is CCWFO. Although CCWFO ranks second, BWEMFO still outperforms it on 17 test functions. For the classic swarm intelligence algorithms PSO and DE, our proposed BWEMFO outperforms them in 23 and 30 test functions, respectively, indicating that the improvement of BWEMFO is effective.

**Fig 3:**
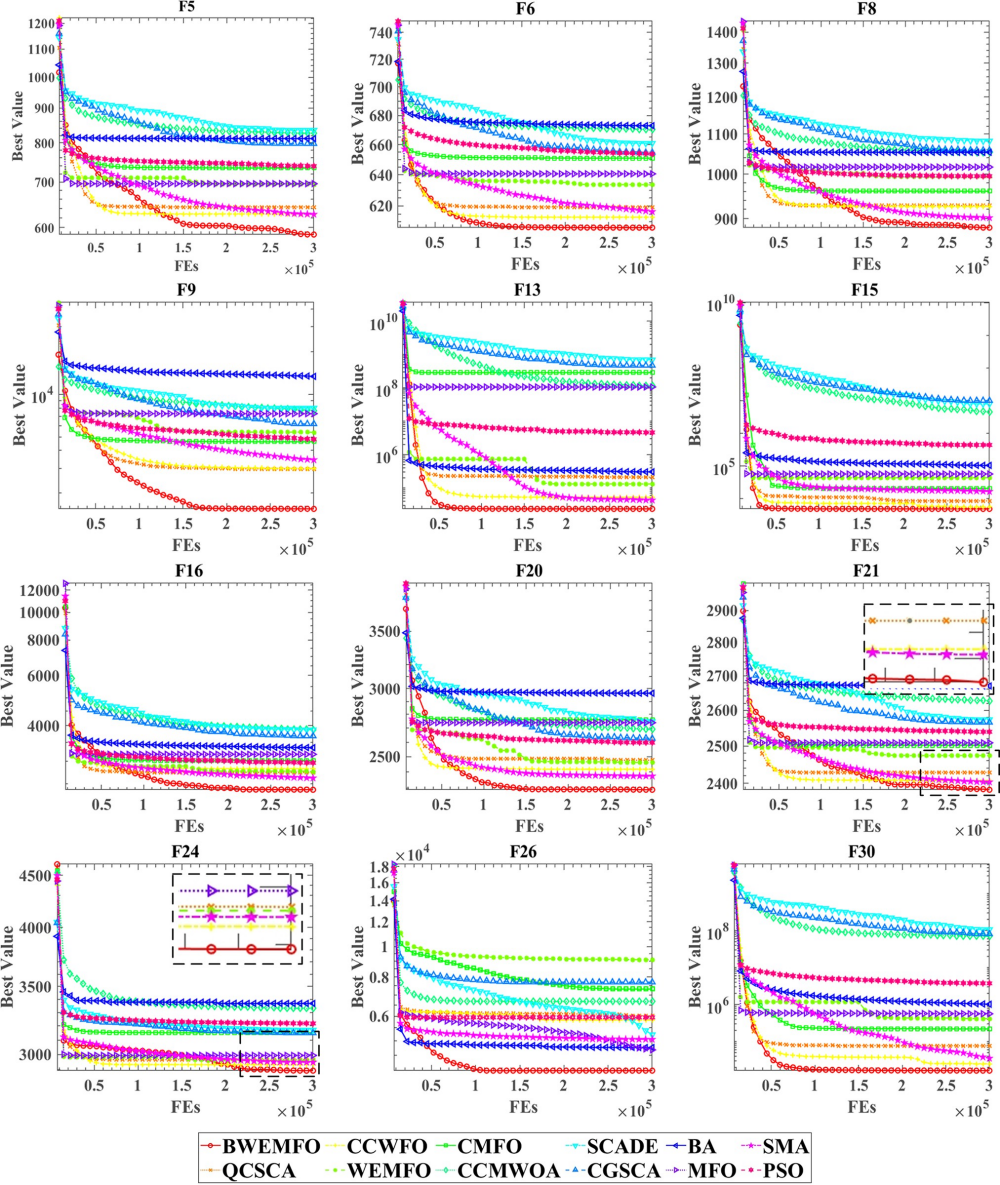
Comparison of convergence curves between BWEMFO and other well-known optimization metheds on IEEE CEC 2017 test funetions.

The convergence curves for BWEMFO and other 12 conventional algorithms are illustrated in Fig 3. More detailed results can be found in Appendix A. The red curve represents BWEMFO. Notably, when other conventional algorithms get trapped in local optima, BWEMFO exhibits a distinct capability to identify and navigate out of these local optima during the latter part. This highlights the robust search ability of BWEMFO. The combination of data presented in tables and the convergence curves leads to the conclusion that BWEMFO, leveraging the synergistic effects of GB, ES and WS, significantly enhances the search capabilities of MFO. As a result, BWEMFO stands out among its competitors.

### 4.4. Application to medical diagnosis

The pivotal function of intelligent optimization algorithms lies in addressing real-world problems [37]. In this section, the BWEMFO method was integrated with KELM, a prominent model in the realm of machine learning. BWEMFO was employed to optimize the two kernel parameters of KELM, leading to the introduction of a novel model termed BWEMFO-KELM. Subsequently, the BWEMFO-KELM model will undergo comparative analysis with four other well-established classifier models. The foundational data for this experiment comprises authentic medical data sourced from both the UCI machine learning repository [38].

KELM is an extended version of Extreme Learning Machine (ELM), which effectively solves nonlinear problems by introducing a kernel function that implicitly maps the input data to a high-dimensional feature space. KELM inherits the high efficiency and ease of use of ELM, and at the same time, enhances its modelling capability for complex nonlinear problems. The KELM used in this study employs a Sigmoid kernel function, where the α and c parameters are the two sum parameters being optimized. α is the weight factor of the kernel function, which controls the scaling of the input vectors. c parameter is the bias term, which controls the translation of the Sigmoid function. To ensure the best performance of the KELM model, the choice of α and c is particularly important. α is usually set in the range of 0.001 ≤ α ≤ 10. Larger values of α may lead to over-sensitivity of the kernel function and thus over-fitting of the model, while smaller values may lead to insufficient generalization of the model. c is usually set in the range of -5 ≤ c ≤ 5. The bias term c influences the center position of the sigmoid function, and adjusting c can better adapt to the distribution of the data.

In this experimental evaluation using medical data, the parameter configurations for both BWEMFO-KELM and MFO-KELM remain consistent. Specifically, the population size and maximum iterations are set at 10 and 50. As a reference model in this study, SVM’s parameters C and γ are also defined within the interval [2^-5, 2^5]. Furthermore, a Gaussian kernel is employed for optimization, with its coefficients determined through grid search. The implementation of SVM utilizes LIBSVM [100]. The KNN model is configured with a single nearest neighbor and employs Euclidean distance. The CART model is instantiated using the classification tree with default parameters. The BP model is realized through the Levenberg-Marquardt algorithm available in the MATLAB toolbox, with 8 neurons in the hidden layer and a mean square error target of 0.001. Due to the inability to directly apply the models to the actual medical data, preprocessing steps primarily involve standardization and normalization within the range [–1, 1]. Standardizing the data enhances uniformity, ensuring compatibility with all experimental models and facilitating the demonstration of the proposed algorithm’s advantages. Ten-fold cross-validation is employed for classification on the standardized data. This methodology involves dividing the dataset into ten equal parts, with nine parts utilized for training in each iteration and the remaining part reserved for validation to assess model accuracy. Utilizing the 10-fold cross-validation approach enhances efficiency in utilizing limited data resources for experimentation.

#### 4.4.1. Metrics of the classification performance

In this study, to showcase the superior performance of the proposed BWEMFO-KELM model in real-world medical diagnosis and classification tasks, we employ *Sensitivity*, *Specificity*, accuracy (*ACC*), and Matthews correlation coefficient (*MCC*) as evaluation metrics. These metrics are abstractly defined as follows:

$$ACC=\frac{TP+TN}{TP+FP+FN+TN}\times 100\%$$ (14)

$$Sensitivity=\frac{TP}{TP+FN}\times 100\%$$ (15)

$$Specificity=\frac{TN}{FP+TN}\times 100\%$$ (16)

$$MCC=\frac{TP\times TN-FP\times FN}{\sqrt{\left(TP+FP)\times (TP+FN)\times (TN+FP)\times (TN+FN\right)}}\times 100\%$$ (17)

where *FN* represents instances classified as false negatives, *FP* denotes instances classified as false positives, *TP* signifies instances classified as true positives, and *TN* indicates instances classified as true negatives.

#### 4.4.2. Breast cancer dataset

The Breast Cancer dataset, originally introduced by Dr. Wolberg [39], is a frequently utilized medical dataset containing 699 instances, each characterized by 10 attributes. Table 6 presents the outcomes observed across tenfold cross-validation iterations. The experimental findings for BWEMFO-KELM are delineated as follows: ACC achieves 98.17%, sensitivity attains 96.93%, specificity reaches 97.50%, and the Matthews correlation coefficient (MCC) stands at 94.26%.

**Table 6:** Experimental results of BWEMFO-KELM with other methods on breast cancer dataset.

Models	Indicator	Mean	std	1	2	3	4	5	6	7	8	9	10
KNN	ACC	0.9557	0.0206	0.9714	0.9571	0.9855	0.9429	0.9286	0.9571	0.9143	0.9714	0.9714	0.9571
CART		0.9428	0.0111	0.9714	0.9286	0.9420	0.9429	0.9429	0.9286	0.9429	0.9429	0.9429	0.9429
BP		0.9371	0.0339	0.9714	0.8857	1.0000	0.9000	0.9143	0.9429	0.9714	0.9429	0.9286	0.9143
SVM		0.9671	0.0239	1.0000	0.9286	0.9855	0.9571	0.9714	0.9571	0.9714	1.0000	0.9714	0.9286
MFO-KELM		0.9657	0.0249	1.0000	0.9429	1.0000	0.9429	0.9571	0.9571	0.9571	1.0000	0.9714	0.9286
BWEMFO-KELM		0.9817	0.0129	1.0000	0.9829	0.9855	0.9729	0.9571	0.9871	0.9714	1.0000	0.9714	0.9886
KNN	Sensitivity	0.9693	0.0240	0.9556	0.9545	1.0000	0.9362	0.9750	0.9362	1.0000	1.0000	0.9796	0.9556
CART		0.9584	0.0243	0.9556	0.9318	1.0000	0.9362	0.9750	0.9149	0.9783	0.9778	0.9592	0.9556
BP		0.9541	0.0365	0.9778	0.9091	1.0000	0.8723	0.9750	0.9362	0.9783	0.9778	0.9592	0.9556
SVM		0.9693	0.0285	1.0000	0.9091	0.9800	0.9574	0.9750	0.9362	1.0000	1.0000	0.9796	0.9556
MFO-KELM		0.9714	0.0277	1.0000	0.9318	1.0000	0.9362	0.9750	0.9362	1.0000	1.0000	0.9796	0.9556
BWEMFO-KELM		0.9894	0.0090	1.0000	0.9918	0.9800	0.9962	0.9750	0.9862	1.0000	1.0000	0.9796	0.9856
KNN	Specificity	0.9314	0.0705	1.0000	0.9615	0.9474	0.9565	0.8667	1.0000	0.7500	0.9200	0.9524	0.9600
CART		0.9105	0.0543	1.0000	0.9231	0.7895	0.9565	0.9000	0.9565	0.8750	0.8800	0.9048	0.9200
BP		0.9088	0.0598	0.9600	0.8462	1.0000	0.9565	0.8333	0.9565	0.9583	0.8800	0.8571	0.8400
SVM		0.9634	0.0383	1.0000	0.9615	1.0000	0.9565	0.9667	1.0000	0.9167	1.0000	0.9524	0.8800
MFO-KELM		0.9559	0.0454	1.0000	0.9615	1.0000	0.9565	0.9333	1.0000	0.8750	1.0000	0.9524	0.8800
BWEMFO-KELM		0.9750	0.0265	1.0000	0.9615	1.0000	0.9565	0.9833	1.0000	0.9167	1.0000	0.9524	0.9800
KNN	MCC	0.9047	0.0426	0.9406	0.9094	0.9637	0.8751	0.8557	0.9100	0.8145	0.9385	0.9320	0.9079
CART		0.8737	0.0252	0.9406	0.8486	0.8550	0.8751	0.8839	0.8471	0.8724	0.8751	0.8639	0.8756
BP		0.8642	0.0717	0.9378	0.7552	1.0000	0.7947	0.8279	0.8751	0.9366	0.8751	0.8281	0.8116
SVM		0.9288	0.0505	1.0000	0.8540	0.9649	0.9044	0.9417	0.9100	0.9373	1.0000	0.9320	0.8435
MFO-KELM		0.9260	0.0536	1.0000	0.8811	1.0000	0.8751	0.9125	0.9100	0.9063	1.0000	0.9320	0.8435
BWEMFO-KELM		0.9426	0.0392	1.0000	0.9811	0.9649	0.8951	0.9125	0.9100	0.9373	1.0000	0.9320	0.8935

**Fig 4:**
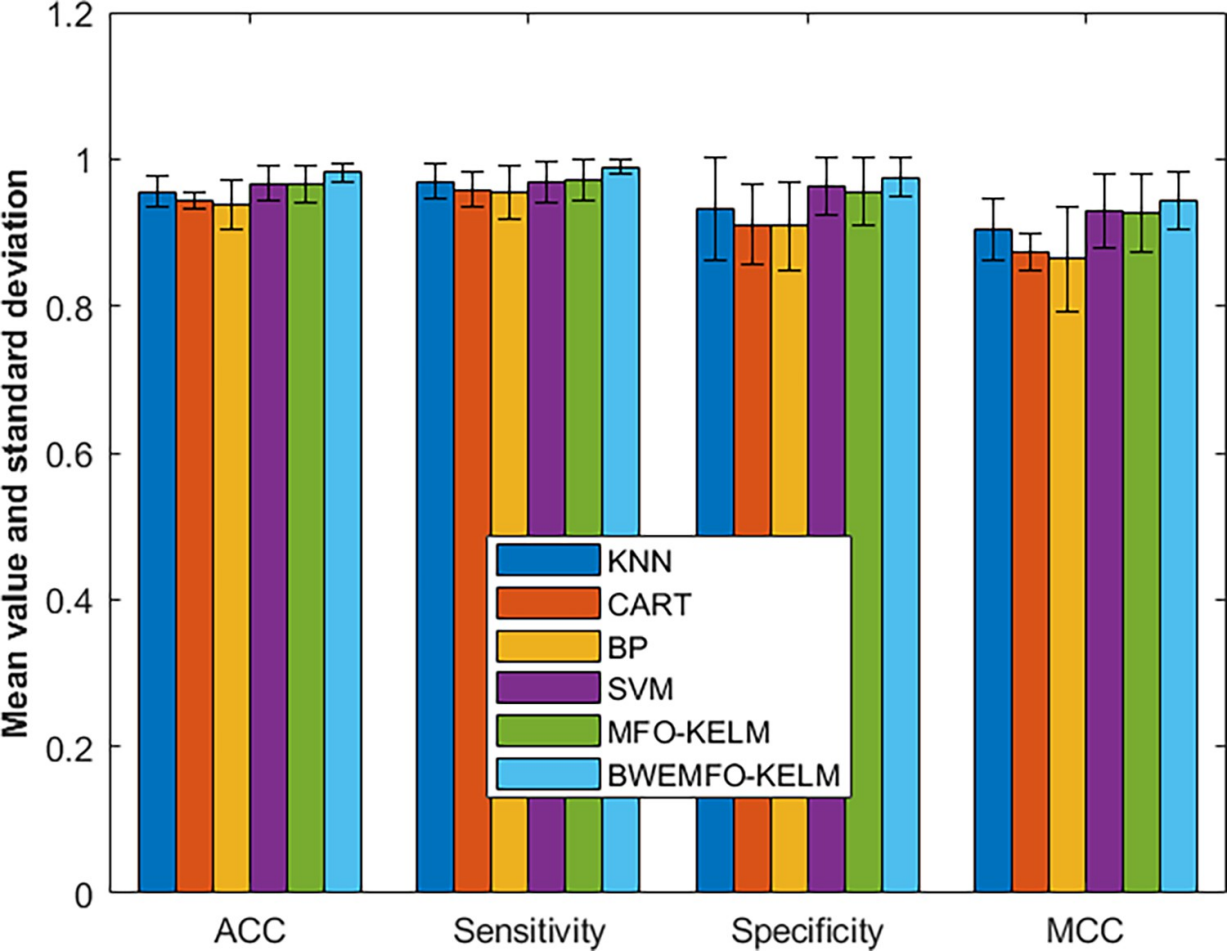
Experimental results of BWEMMO-KELM with other methods on breast cancer dataset.

In Fig 4, we present a graphical representation of the experimental outcomes, facilitating a visual assessment of the results. As depicted in the graph, BWEMFO-KELM demonstrates superior performance across all four metrics. Particularly noteworthy is its leading position in ACC, surpassing SVM, which ranks second, with BP exhibiting the least favorable performance. While SVM typically exhibits strengths in classifying small sample sizes, in this experiment, the four metrics for SVM fall short of those achieved by BWEMFO-KELM, highlighting the performance advantages of the latter. Moreover, BWEMFO-KELM outperforms MFO-KELM across all four indicators, underscoring the efficacy of BWEMFO within the MFO framework.

#### 4.4.3. Colorectal cancer datasets

The colorectal cancer datasets, as documented in Forsyth’s report [40], is a widely employed medical dataset containing 345 instances, each characterized by 7 attributes. Table 7 presents the experimental outcomes for the four indicators.

**Table 7:** Experimental results of BWEMFO-KELM with other methods on colorectal cancer datasets.

Models	Indicator	Mean	std	1	2	3	4	5	6	7	8	9	10
KNN	ACC	0.6178	0.0735	0.6286	0.6471	0.6471	0.5143	0.6857	0.6571	0.5882	0.4571	0.7059	0.6471
CART		0.6415	0.0935	0.5714	0.6176	0.7353	0.6571	0.6571	0.5143	0.7353	0.4857	0.6471	0.7941
BP		0.6554	0.0847	0.7143	0.7353	0.6765	0.6286	0.7143	0.4571	0.6176	0.6571	0.7647	0.5882
SVM		0.7279	0.0834	0.7714	0.7059	0.8235	0.6571	0.8286	0.5429	0.6765	0.7143	0.7941	0.7647
MFO-KELM		0.7279	0.0835	0.8286	0.6765	0.7941	0.6286	0.7714	0.5714	0.6765	0.7143	0.8235	0.7941
BWEMFO-KELM		0.8022	0.0342	0.8000	0.7765	0.7941	0.7571	0.8429	0.7714	0.8765	0.7857	0.8235	0.7941
KNN	Sensitivity	0.5490	0.0840	0.5882	0.5000	0.5333	0.5000	0.5833	0.5000	0.4667	0.4375	0.7143	0.6667
CART		0.5513	0.1699	0.5294	0.5000	0.6000	0.5000	0.7500	0.2500	0.6667	0.3125	0.5714	0.8333
BP		0.5490	0.1193	0.5294	0.5714	0.6667	0.5714	0.6667	0.2500	0.4667	0.6250	0.6429	0.5000
SVM		0.5941	0.1498	0.7059	0.5000	0.7333	0.5000	0.7500	0.2500	0.6000	0.6875	0.7143	0.5000
MFO-KELM		0.6020	0.1558	0.7059	0.4286	0.8000	0.5000	0.6667	0.2500	0.6000	0.6875	0.7143	0.6667
BWEMFO-KELM		0.6574	0.0775	0.7059	0.6286	0.8000	0.5000	0.5833	0.6500	0.7000	0.6250	0.7143	0.6667
KNN	Specificity	0.6700	0.0960	0.6667	0.7500	0.7368	0.5238	0.7391	0.7895	0.6842	0.4737	0.7000	0.6364
CART		0.7154	0.0756	0.6111	0.7000	0.8421	0.7619	0.6087	0.7368	0.7895	0.6316	0.7000	0.7727
BP		0.7368	0.0897	0.8889	0.8500	0.6842	0.6667	0.7391	0.6316	0.7368	0.6842	0.8500	0.6364
SVM		0.8232	0.0600	0.8333	0.8500	0.8947	0.7619	0.8696	0.7895	0.7368	0.7368	0.8500	0.9091
MFO-KELM		0.8204	0.0715	0.9444	0.8500	0.7895	0.7143	0.8261	0.8421	0.7368	0.7368	0.9000	0.8636
BWEMFO-KELM		0.8896	0.0576	0.8889	0.8500	0.7895	0.9619	0.8261	0.8421	0.9368	0.9368	0.9000	0.9636
KNN	MCC	0.2197	0.1417	0.2557	0.2575	0.2760	0.0233	0.3168	0.3038	0.1542	-0.0885	0.4085	0.2901
CART		0.2655	0.1963	0.1410	0.2025	0.4594	0.2703	0.3407	-0.0150	0.4602	-0.0587	0.2714	0.5833
BP		0.2905	0.1786	0.4503	0.4433	0.3490	0.2357	0.3932	-0.1271	0.2114	0.3092	0.5076	0.1324
SVM		0.4298	0.1726	0.5446	0.3780	0.6417	0.2703	0.6196	0.0468	0.3398	0.4243	0.5715	0.4609
MFO-KELM		0.4333	0.1754	0.6727	0.3108	0.5864	0.2173	0.4928	0.1147	0.3398	0.4243	0.6326	0.5417
BWEMFO-KELM		0.5186	0.1640	0.6068	0.6108	0.5864	0.6703	0.4186	0.1147	0.6398	0.3642	0.6326	0.5417

**Fig 5:**
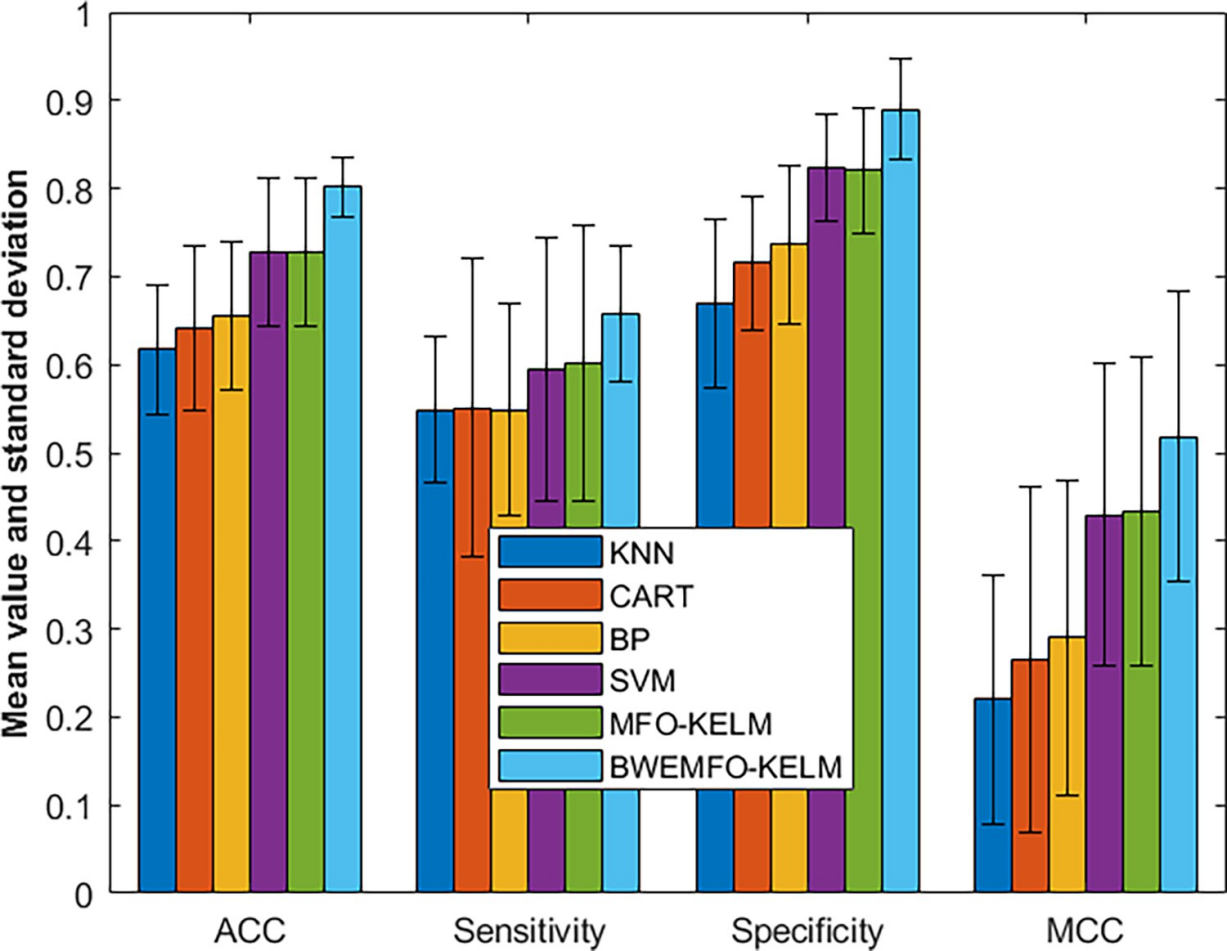
Experimental results of BWEMFO-KELM with other methods on bupa liver dataset.

Fig 5 illustrates the experimental findings, showcasing BWEMFO-KELM’s superior performance in four metrics, where it secures the top rank, markedly outperforming the original MFO-KELM algorithm. This underscores BWEMFO-KELM’s efficacy in effectively classifying positive cases within the dataset, a crucial aspect in medical diagnosis, as it signifies the model’s enhanced ability to discern individuals with the condition. These findings suggest that the new BWEMFO-KELM variant offers notable advantages over the original MFO-KELM and delivers superior performance compared to traditional models.

#### 4.4.4. Mammographic dataset

The mammographic medical dataset [41] is frequently utilized in medical research, comprising 961 instances, each characterized by 6 attributes. Table 8 presents the experimental outcomes of BWEMFO-KELM on this dataset.

**Table 8:** Experimental results of BWEMFO-KELM with other methods on mammographic dataset.

Models	Indicator	Mean	std	1	2	3	4	5	6	7	8	9	10
KNN	ACC	0.7271	0.0298	0.6771	0.7292	0.7500	0.6979	0.7188	0.7396	0.7396	0.7708	0.7604	0.6875
CART		0.7823	0.0230	0.7813	0.7500	0.7708	0.7604	0.7813	0.8229	0.7708	0.7917	0.8229	0.7708
BP		0.8188	0.0330	0.8542	0.7604	0.8125	0.8021	0.8229	0.8333	0.8333	0.8229	0.8750	0.7708
SVM		0.8104	0.0250	0.8125	0.7813	0.8021	0.8125	0.8438	0.8333	0.8542	0.8021	0.7813	0.7813
MFO-KELM		0.8177	0.0248	0.8125	0.7813	0.8125	0.8229	0.8438	0.8333	0.8646	0.8021	0.8229	0.7813
BWEMFO-KELM		0.8405	0.0301	0.8125	0.8813	0.8021	0.8229	0.8333	0.8925	0.8646	0.8125	0.8229	0.8604
KNN	Sensitivity	0.6871	0.0342	0.6977	0.6829	0.7255	0.6579	0.6981	0.7143	0.6818	0.7083	0.7045	0.6000
CART		0.7625	0.0450	0.8140	0.6829	0.7843	0.7368	0.7358	0.8095	0.7727	0.7708	0.8182	0.7000
BP		0.8077	0.0691	0.8837	0.6829	0.8235	0.7632	0.8491	0.8095	0.7955	0.8125	0.9318	0.7250
SVM		0.7974	0.0418	0.8140	0.7317	0.7843	0.7895	0.8868	0.8333	0.8182	0.7708	0.7955	0.7500
MFO-KELM		0.8262	0.0514	0.8605	0.7073	0.8627	0.8158	0.8868	0.8095	0.8409	0.7917	0.8864	0.8000
BWEMFO-KELM		0.8528	0.0334	0.8605	0.8073	0.8235	0.8858	0.8679	0.8895	0.8409	0.7917	0.8864	0.8750
KNN	Specificity	0.7609	0.0451	0.6604	0.7636	0.7778	0.7241	0.7442	0.7593	0.7885	0.8333	0.8077	0.7500
CART		0.7987	0.0306	0.7547	0.8000	0.7556	0.7759	0.8372	0.8333	0.7692	0.8125	0.8269	0.8214
BP		0.8248	0.0218	0.8302	0.8182	0.8000	0.8276	0.7907	0.8519	0.8654	0.8333	0.8269	0.8036
SVM		0.8194	0.0289	0.8113	0.8182	0.8222	0.8276	0.7907	0.8333	0.8846	0.8333	0.7692	0.8036
MFO-KELM		0.8070	0.0404	0.7736	0.8364	0.7556	0.8276	0.7907	0.8519	0.8846	0.8125	0.7692	0.7679
BWEMFO-KELM		0.8488	0.0299	0.8736	0.8364	0.8778	0.8276	0.8907	0.8148	0.8846	0.8333	0.7992	0.8500
KNN	MCC	0.4484	0.0640	0.3561	0.4466	0.5024	0.3777	0.4399	0.4724	0.4737	0.5459	0.5160	0.3528
CART		0.5605	0.0496	0.5656	0.4864	0.5399	0.5069	0.5704	0.6413	0.5405	0.5838	0.6441	0.5257
BP		0.6327	0.0722	0.7102	0.5069	0.6235	0.5882	0.6413	0.6614	0.6637	0.6460	0.7568	0.5286
SVM		0.6165	0.0513	0.6231	0.5517	0.6054	0.6121	0.6834	0.6637	0.7059	0.6054	0.5628	0.5517
MFO-KELM		0.6332	0.0506	0.6307	0.5499	0.6238	0.6361	0.6834	0.6614	0.7270	0.6043	0.6546	0.5610
BWEMFO-KELM		0.6628	0.0490	0.6307	0.7499	0.6022	0.6361	0.6621	0.7216	0.7270	0.6255	0.6546	0.6187

**Fig 6:**
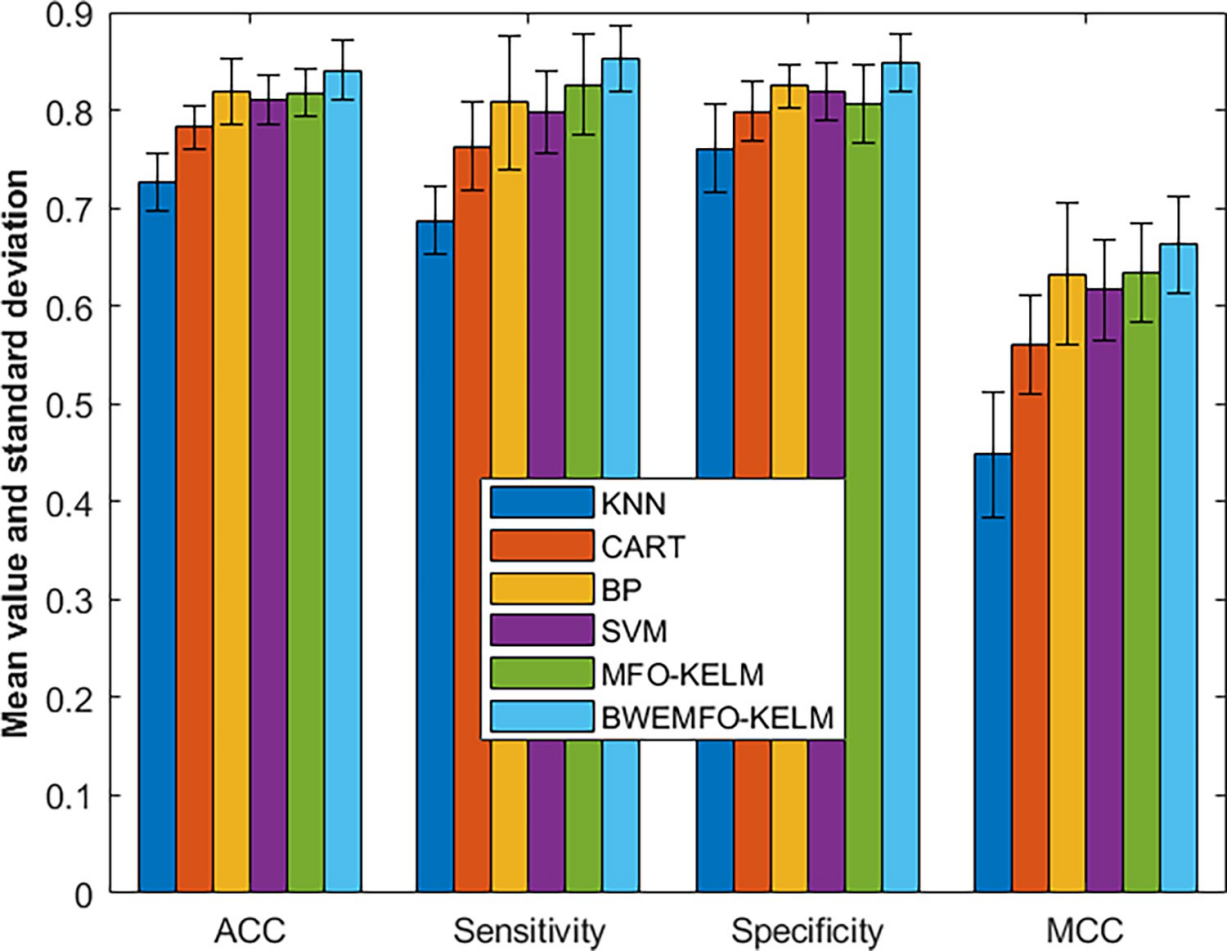
Experimental results of BWEMFO-KELM with other methods on mammographic dataset.

In Fig 6, we present the experimental outcomes, where BWEMFO-KELM emerges as the top performer across the four measurement factors, securing the first rank and demonstrating significant improvement over the original MFO-KELM model. These experimental findings underscore the enhanced advantages of the new BWEMFO-KELM model over its predecessor, particularly evident in sensitivity, in comparison to other traditional models.

## 5. Discussion

This paper introduces a novel enhanced MFO algorithm (BWEMFO) designed to improve algorithmic performance. This improvement is achieved by incorporating a Gaussian barebone mechanism, a wormhole strategy, and an elimination strategy into the MFO. The Gaussian barebone mechanism expedites algorithm convergence by employing a reduced random rate and an increased step size. Concurrently, the wormhole strategy and elimination strategy augment global exploration by fostering information exchange among individuals, thereby heightening the probability of evading local optima.

In BWEMFO, the wormhole strategy simulates the concept of wormholes to create a "jump" mechanism in the search space, enabling the algorithm to directly leap from the current solution’s local region to other distant areas. This strategy facilitates the exploration of more regions within the search space and helps avoid premature convergence to local optima, thereby enhancing the algorithm’s global search capability. The wormhole strategy allows the algorithm to quickly jump during the global search phase, while enabling precise optimization in the local search phase, effectively balancing global exploration and local exploitation. Through this strategy, BWEMFO is better able to locate high-quality global optima in the search space.

The elimination strategy, which periodically removes individuals with poor fitness, helps maintain population diversity and prevents premature convergence of individuals to local optima. By eliminating low-performing individuals, the strategy allows better individuals more opportunities for reproduction and update, thereby improving the overall population fitness and the search ability of the algorithm. The elimination strategy removes low-quality solutions in a timely manner, reducing computational resource wastage and accelerating the convergence speed of the algorithm. This is particularly important when solving complex optimization problems and contributes to improving computational efficiency.

The Gaussian barebone mechanism adjusts the search direction and step size by introducing a Gaussian distribution, allowing for more refined search behavior. This mechanism effectively guides the solutions toward better regions, improving the precision of local search. In traditional MFO algorithms, local search is prone to getting trapped in local optima, especially in high-dimensional and complex problems. By incorporating the Gaussian barebone mechanism, BWEMFO can more flexibly adjust the step size and search range, avoiding over-reliance on specific regions and thus enhancing the algorithm’s global search capability.

By combining the wormhole strategy, elimination strategy, and Gaussian barebone mechanism in MFO, these three strategies complement and synergize with each other. The wormhole strategy offers broad global search capability, the elimination strategy enhances population diversity and accelerates convergence, while the Gaussian barebone mechanism refines local search precision. Overall, the integration of these strategies significantly improves the optimization performance of BWEMFO, particularly in tackling complex, high-dimensional optimization problems. This combination ensures a better balance between solution quality and algorithm efficiency, while maintaining search diversity.

## 6. Conclusions

In the present study, we introduce BWEMFO, which incorporates a Gaussian barebone mechanism, a wormhole strategy, and an elimination strategy to enhance the efficacy of the moth flame optimization. The Gaussian barebone mechanism expedites algorithm convergence by employing a reduced random rate and an increased step size. Concurrently, the wormhole strategy and elimination strategy augment global exploration by fostering information exchange among individuals, thereby heightening the probability of evading local optima.

We systematically compare the proposed BWEMFO with algorithms, advanced algorithms, and MFO variants utilizing IEEE CEC 2017 benchmark functions. Empirical results illustrate that BWEMFO surpasses other optimizers in both convergence speed and solution accuracy across diverse benchmark functions. Furthermore, the proposed BWEMFO-KELM performs diagnostic and predictive experiments on three medical datasets: breast cancer dataset, colorectal cancer datasets and mammographic dataset. After comparing with other five machine learning methods on four evaluation metrics, the experimental results show that the proposed BWEMFO-KELM model has higher diagnostic accuracy and reliability. In future endeavors, our focus will be on further enhancing BWEMFO to mitigate algorithmic time costs while concurrently improving optimization performance. Additionally, we plan to extend the application of BWEMFO to diverse scenarios. Although this study has achieved satisfactory experimental results, it still has some limitations. Specifically, in the enhanced MFO, we should consider the potential of eliminated individuals during each iteration, as some of these may be promising candidates. Additionally, the introduction of Gaussian barebone mechanism and wormhole strategy may increase the computational complexity of the algorithm, leading to longer execution times in certain cases, particularly in high-dimensional problems. The performance of Gaussian barebone mechanism and wormhole strategy often depends on the parameter settings, and inappropriate parameters may adversely affect the convergence speed and search capability of the algorithm. In future work, we will focus on addressing these issues to further improve the algorithm’s performance. Different population sizes may have an impact on the effectiveness of the algorithm, and we will also pay attention to this in future work to improve the algorithm’s performance.

## Supporting information

S1 Appendix This section presents the complete experimental results of the proposed BWEMFO on the IEEE CEC 2017 test dataset, including convergence curve graphs on 30 test functions. (DOCX)

S1 Data This section provides medical data tables used in the experiment. (RAR)
