# Supplementary material for: Gaussian barebone mechanism and wormhole strategy enhanced moth flame optimization for global optimization and medical diagnostics
Source: PLoS One. 2025 Jan 16;20(1):e0317224. doi: 10.1371/journal.pone.0317224 (PMC11737686; doi:10.1371/journal.pone.0317224)
Supplement: S1 Appendix — (DOCX) [file pone.0317224.s001.docx]

1. Appendix

4.3. Comparison with other algorithms


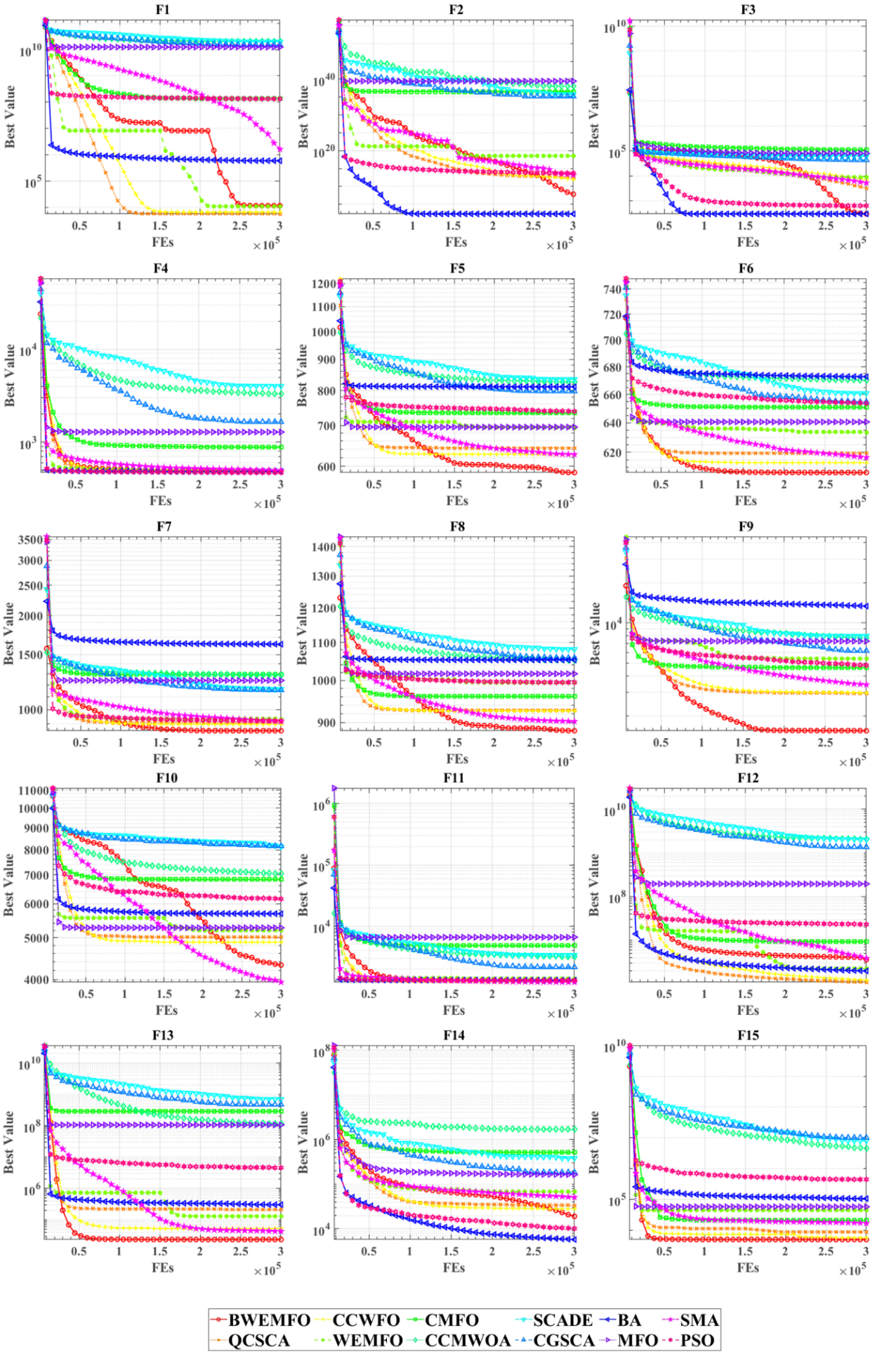


**Fig 7.** Comparison of convergence curves between BWEMFO and other well-known optimization methods on IEEE CEC 2017 test functions

**
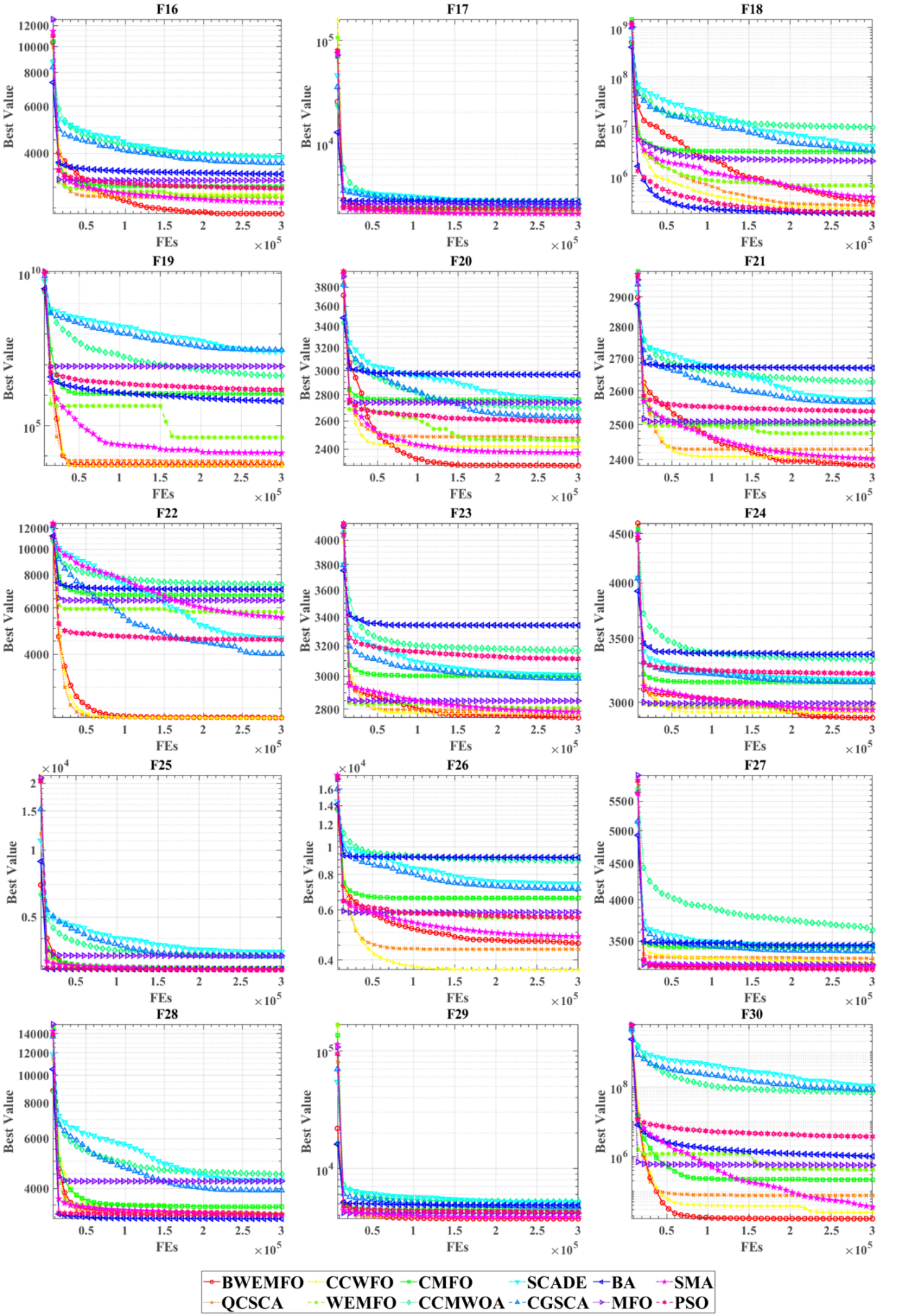
**

**Fig 8.** Comparison of convergence curves between BWEMFO and other well-known optimization methods on IEEE CEC 2017 test functions
